# Toward the Development of Potentially Healthy Low-Energy-Density Snacks for Children Based on Pseudocereal and Pulse Flours

**DOI:** 10.3390/foods12152873

**Published:** 2023-07-28

**Authors:** Maria Eugenia Martín-Esparza, María Dolores Raigón, María Dolores García-Martínez, Ana Albors

**Affiliations:** 1 Institute of Food Engineering for Development, Food Technology Department, Universitat Politècnica de València, 46022 Valencia, Spain; analbors@tal.upv.es; 2 Institute for the Preservation and Improvement of Valencian Agro-Diversity, Food Chemistry Department, Universitat Politècnica de València, 46022 Valencia, Spain; mdraigon@qim.upv.es (M.D.R.); magarma8@gmail.com (M.D.G.-M.)

**Keywords:** quinoa, chickpea, inulin, childhood obesity, gluten-free

## Abstract

The main objective of this study was the development of gluten-free cracker-type snacks with a balanced supply of essential amino acids, a lower glycemic index, and a lower caloric intake that were sensorially acceptable. For this purpose, chickpea flour was replaced by quinoa (10, 20, 30, 40, and 50%) and the fat was partially (75%) replaced by chicory inulin. The flours were characterized in terms of their proximate composition, individual mineral content, particle size distribution, and functional properties. The parameters analyzed for the crackers, once baked, were the water content, water activity, weight, dimensions, color, and texture. A sensory analysis was performed as well, using the formulations containing 50% chickpea flour and 50% quinoa flour (g/100 g flour), with and without inulin, as well as those made with 100% chickpea flour. From the analysis of the raw flours, it can be concluded that snack products developed from them could be a nutritive option for children, in terms of the protein, magnesium, and fiber content. The functional properties revealed that both flours are suitable for producing doughs and baked products. The obtained results indicate that snacks made with 50% quinoa flour (g/100 g flour) and 75% chicory inulin (g/100 g high oleic sunflower oil) could be an interesting alternative for children as, in addition to offering a very interesting nutritional contribution, the energy intake from fat is reduced by 57%.

## 1. Introduction

The World Health Organization has determined that in 2016, worldwide, more than 39% of the adult male population, and 40% of the female population were overweight, and that around 13% of the global population suffered from obesity [1]. Moreover, the worldwide prevalence of obesity nearly tripled between 1975 and 2016, dramatically increasing among children and adolescents aged 5–19, from 4% to 18% [1]. Childhood obesity is one of the main public health problems in Europe, and is associated, together with overweight, with numerous health complications in adulthood, such as diabetes and heart disease, as well as psychological effects [2]. Among the factors associated with obesity, those related to eating habits continue to stand out. This is a problem that must be addressed through an individualized therapeutic strategy that includes not only diet and physical activity, but also genetic factors. However, a correct relationship between calorie intake and physical activity continues to be one of the main ways to reduce the risk of overweight from an early age. For this reason, the search for new products attractive to this sector of the population, such as cracker-type snacks, with a satiating capacity, adequate nutritional intake, and lower energy density, can help to achieve this objective.

Cracker-type snacks can be a very interesting option for children, and adults, too, as they are inexpensive, accessible, and ready-to-eat products with a long shelf-life, which can be easily consumed at any time of the day, and which have a pleasant texture and taste. However, the energy consumed from snacking is still often high, especially due to the caloric content of the fats used in the snacks’ formulation. In addition, the nutritional intake, in terms of dietary fiber, vitamins, minerals, and essential amino acids, is often inadequate. It is true that in recent years, the snack market has changed considerably, and a variety of more nutrient-dense snacks can now be found. However, there is still a need to develop appealing snacks which are both high in protein and dietary fiber, and a good source of minerals, while reducing the fat composition and, therefore, the total energy intake [3]. As a reference, an energy requirement of 2000 kcal per day is recommended at the age of 6–9 years, with 20–30% of this energy supplied by fat [4,5]. The use of inulin, a non-digestible dietary fiber typically derived from chicory root, have been found to be an excellent high-level fat replacer, with minimal impact on the sensory properties and consumer acceptance of legume crackers [6]. Inulin interacts with water and, eventually agglomerates, creating a gel network that appears to have the ability to mimic the functions of fat in baked products [7]. Furthermore, inulin has the advantage over other potential fat replacers of possibly helping to achieve fiber content claims. It has promising gut-health properties, due to its prebiotic nature, and may increase the absorption of nutrients such as calcium [6].

To achieve an adequate nutritional profile in the final snack product, it is important to choose the most suitable raw materials, selecting those rich in protein, dietary fiber, slow-digesting carbohydrates, vitamins, and minerals, among other components. In this sense, quinoa and chickpea flours may be excellent candidates and, to our knowledge, the combination of both flours has not been used before to produce cracker-type snacks. Additionally, it seems reasonable to consider other factors that are more related to the availability, crop requirements, and yields, when choosing alternative raw materials. One of the options is to consider crops with a low water demand, and a high resistance to adverse agroclimatic conditions, such as quinoa and chickpeas [8,9]. It is well known that protein intake in the diet is very important, especially (although not only) in childhood, because it is a stage of continuous growth. As a reference, at the age of 6–9 years, a daily intake of 0.89–0.92 g/kg bw/day is recommended [4,5]. However, it is not only the amount of protein ingested that is crucial but, above all, the biological value of these proteins. Compared to true cereals, quinoa has a higher protein content (10.4–17%), with a greater quantity of the eight amino acids considered essential for both children and adults (leucine, iso-leucine, lysine, methionine, phenylalanine, threonine, tryptophan, and valine), and with a certain presence of arginine, histidine, cystine, and tyrosine, which are also essential [10]. Furthermore, quinoa presents a higher lysine content (5–7%), and a much higher level of methionine and cysteine (6–12%), than pulse seeds (2–4%) [11]. Consequently, the genus *Chenopodium* becomes a good source of essential amino acids, approaching the values recommended by [12], and represents a good complement to pulses, such as chickpeas (*Cicer arietinum* L.). In addition, quinoa is also rich in some minerals, especially calcium, magnesium, iron, potassium, and phosphorus, and contains many bioactive compounds, especially phytosterols, polyphenols, saponins, and squalene [13]. A low intake of magnesium has been associated with, among other issues, insulin resistance in obese children [14]. Moreover, during childhood and adolescence, an adequate intake of calcium, phosphorus, and magnesium can contribute to achieving an optimal peak in bone mass, which may help to prevent the development of osteoporosis in the later stages of life [15]. An adequate intake for children aged 4–10 years is 800 mg/day for calcium, 440 mg/day for phosphorus, and 230 mg/day for magnesium [4,5]. In addition, chickpeas (*Cicer arietinum* L.) have a high content of resistant starch and fiber [16]. Some components of chickpeas have shown, in preclinical and clinical trials, several beneficial health effects, such as antioxidant, anticancer, analgesic, anti-inflammatory, and hypocholesteriolemic properties, and also effects related to type II diabetes [17]. Furthermore, chickpeas constitute one of the most consumed pulses in the world, accounting for more than 2.3 million tons per year. 

To the authors’ knowledge, no data have been published on cracker-type snacks based on chickpea flour, as affected by the whole quinoa flour and inulin incorporation. Therefore, this paper aims to: (i) assess the chemical composition, particle size distribution, and functional properties of quinoa and chickpea flours; (ii) evaluate the effect of increasing the substitution levels of the pulse flour with whole quinoa flour up to 50% (*w*/*w*), and replacing 75% (*w*/*w*) of the fat with inulin from chicory, on the physicochemical properties of the final product; and (iii) assessing the consumer acceptance of, the main potential nutritional contribution of, and the energy density reduction in snacks obtained using quinoa at 50% *w*/*w* flour, and inulin at 75% *w*/*w* fat. 

## 2. Materials and Methods

### 2.1. Raw Materials

The quinoa seeds (*Chenopodium quinoa* Willd.) were purchased in a local market, and milled to a fine powder using a blade stainless steel mill (Grindomix GM 200, RETSCH GmbH, Hann, Germany) at 10,000 rpm, over 20 s. The quinoa powder was sieved through a 50 mesh, to obtain wholemeal quinoa flour (QF). Chickpea flour (CPF) and inulin from chicory root (El Amasadero, Málaga, Spain), high oleic sunflower oil (Olis Bargalló, Barcelona, Spain), baking powder (sodium bicarbonate E-500, disodium diphosphate E-450; Royal, Madrid, Spain), soy lecithin (Innovative cooking S.L., Madrid, Spain), water from the drinking-water supply laboratory, and salt (Hacendado S.A., Tavernes Blanques, Spain) were also used in the preparation of the cracker-type snacks.

### 2.2. Flour Characterization

#### 2.2.1. Proximate Composition and Energy Intake

The CPF and QF were analyzed for their water, protein, fat, crude fiber, and ash content, according to the official AOAC methods 984.25, 984.13, 983.23, 991.43, and 923.03, respectively [18]. The digestible carbohydrates were determined by difference (100 minus the percentage of the estimated proximate chemical composition). Three replicates were carried out for each analysis. The energy intake was calculated by multiplying the grams of fat by 9 kcal, and the grams of protein and carbohydrate by 4 kcal.

#### 2.2.2. Mineral Composition

The samples were subjected to digestion, in accordance with method AOAC 985.35 [18]. The samples were calcined in a Carbolite CWF 1100 chamber furnace at 550 °C, and the ashes were dissolved using concentrated HCl, until a 2% HCl solution was obtained. The calibration curves were established using the working standards for each element. The analytical curves were obtained with a linear response for the selected concentration ranges. Mineral analysis was performed, using atomic absorption spectroscopy (AAS), in a THERMO elemental AA series spectrometer (spectrophotometer) and hollow cathode lamps for each element, except for phosphorus, which was analyzed using colorimetry [19].

#### 2.2.3. Particle Size Analysis

The particle size distribution (PSD) of both the CPF and QF was determined by applying the laser diffraction method and Mie theory, following the ISO13320 normative [20]. A laser diffractometer (Mastersizer 2000, Malvern Instruments Ltd., Worcestershire, UK), equipped with a PS 65 (dry sample) or wet sample dispersion unit (Malvern Instruments Ltd., Hydro 2000 MU, Malvern, UK) was employed to perform, respectively, the dry and wet analyses. The particle refraction and absorption indexes were 1.52 and 0.1, respectively, and the water refraction index was 1.03. The distributions were made in triplicate, and for each sample, and 10–20 g of flour was used. Size distribution was quantified as the relative volume of particles in size bands, presented as size distribution curves (Malvern MasterSizer Micro software v 5.40). The PSD parameters recorded included the largest particle size d(0.9), the mean particle volume d(0.5), the smallest particle size d(0.1), and the mean particle diameter/volume mean diameter (D[3,4]). The Span value, or the measurement of the width of the size distribution, calculated from the values of the standard percentiles, was complementarily reported. The wider the particle-size distribution, the bigger the Span becomes. The fundamental size distribution, using the laser diffraction method, is expressed in terms of the equivalent spheres (the results are volume-based), so the number distributions should only be considered as a guide to the volume distribution.

#### 2.2.4. Functional Properties

The functional properties of both CPF and QF were determined, as follows. The water-holding capacity (WHC) was determined using the modified methods from [21,22]. The fat adsorption capacity (FAC) was determined according to [23]. The foam capacity (FC) and foam stability (FS) were determined as described by [24,25].

### 2.3. Experimental Design and Processing of Cracker-Type Snacks

The experimental design was performed considering two factors: the incorporation of quinoa flour at five levels of substitution in place of chickpea flour (0, 10, 20, 30, 40, and 50%), and inulin at two substitution levels (absence, and 75% (g/g) substitution) of sunflower oil. Table 1 summarizes the tested formulations. All the doughs contained 61% flour, 26.05% water, 0.25% salt, 0.2% baking powder, and 0.5% soy lecithin. For the formulations without inulin, the amount of oil was 12%, while for those with inulin, the amount of oil decreased to 3%. According to the literature [6,26], a 75% substitution of sunflower oil by inulin in the cracker formulation would allow us to obtain a product with similar rheological characteristics and an acceptable taste [27], as well as a considerable reduction in the energy density of the final product.

Snacks were prepared in an electric cooking device (Thermomix TM-31, Vorwerk Spain M.S.L., Madrid, Spain) in three steps; the solid and liquid components were weighed (PFB 300-3, Kem & SohnGmbH, Balingen, Germany) and separately mixed. Firstly, once the flours were sieved, to avoid agglomeration and facilitate their subsequent hydration, they were mixed with the salt and the baking powder for 30 s at 200 rpm. Secondly, the oil and the soy lecithin were mixed for 15 s at 300 rpm and, after the water was added, they were emulsified for 2 min at 1100 rpm. Thirdly, the solids were incorporated into the emulsion, and the resulting blends were kneaded together for 3 min at 300 rpm. The formed doughs were rested for 30 min at 4 °C inside a plastic bag, in order to enable sample relaxation, avoiding dehydration. Afterwards, sheets (1.50 ± 0.03 mm thick) were formed using a domestic pasta-making machine (Simplex 152 SP150, Imperia, Italy), coupled with a specific motor (A2500, Imperia, Italy). The final shape of the snacks was obtained by cutting the sheets with a square mold of 4 × 4 cm. The resulting doughs were baked at 165 °C for 10 min in a preheated oven, and left to cool for 15 min in a cool and dry place at room temperature. Finally, the samples were packed in paper bags until further analysis. When inulin is used, it is required a prior dissolution in water (1:1 ratio by weight); therefore, the amount of water used for this purpose has been considered as a part of the total amount of water included in the corresponding formulations. Continuous agitation using a magnetic stirrer (ARE, Velp Scientifica, Inc., Deer Park, NY, USA) for 5 min at medium speed (700 rpm) was used to reach a complete dissolution of the inulin powder. Subsequently, this inulin solution was incorporated into the mixture of oil with soy lecithin and, together with the rest of the water that formed part of the formulations, the emulsion was formed as described earlier. The rest of the process, both before and after this step, was not altered.

### 2.4. Analysis of Cracker-Type Snacks

#### 2.4.1. Physicochemical Analysis

The water content was analyzed according to the AACC method 44-40 [28]: approximately, 5 g of sample (Kern & Sohn GmbH, PFB 300-3, Balingon, Germany) was submitted to 130 ± 1 °C in a vacuum oven, until it reached a constant weight (Selecta, Hotcold B, Barcelona, Spain). AquaLab Series 4 TEV equipment (Decagon Devices, Inc., Pullman, WA, USA) was used to measure water activity (a_w_). The analysis was performed in triplicate for each formulation. Weight was measured using an analytical balance with 0.0001 g precision (Kern & Sohn GmbH, PFB 300-3, Balingen, Germany). Product dimensions (thickness and diameter) were assessed according to AACC method 10–50.05 D [28], using a caliper (PCE Ibérica S.L., PCE-DCP 200N, Albacete, Spain) with a sensitivity of 0.02 mm. This determination was made in sextuplicate.

The proximate composition of the snacks was estimated by considering the chemical composition of the raw materials, and the mass loss during baking, corresponding to the water evaporation.

#### 2.4.2. Snack Firmness

Snack firmness was determined using a TA.XT2 Texture Analyzer (Stable Micro Systems, Godalming, Surrey, UK), through a three point flexion test. The conditions were: speed pre-test 1 mm/s, speed during test 3 mm/s, speed post-test 10 mm/s, distance 5 mm, and an activation force of 0.049 N. The length between the supports was 2 cm. The test was conducted in sextuplicate.

#### 2.4.3. Optical Properties

The optical properties of the cracker-type snacks were determined in triplicate by measuring the reflection spectra of the samples from 400 to 700 nm of wavelength, using a MINOLTA spectrocolorimeter (model CM-3600d, Minolta CO., Tokyo, Japan). The CIEL*a*b* color coordinates were obtained from the reflectance of an infinitely thick layer of the sample, by considering the illuminant D65 and observer 10°. The instrument was calibrated with a white reference tile (L = 97.10, a = −4.88, b = 7.04) before the measurements. According to the CIE color system (International Commission on Illumination), L* represents the brightness on a scale of 0 (black) to 100 (white), a* represents a scale of +50 (red) to −50 (green), and b* represents a scale of +50 (yellow) to −50 (blue). The psychometric coordinates of the chroma (C_ab_*) and hue angle (h_ab_*) were also determined. The browning index (BI) was calculated from the measured L*, a* and b* values, using Equation (1) [29].

(1) $$BI=\frac{100\cdot \left(x-0.31\right)}{0.17}$$

where $x=\frac{a^{*}\;+\;1.79\;\cdot \;L^{x}}{5.645\;\cdot \;L^{x}\;+\;a^{*}-3.012\;\cdot \;b^{*}}$.

#### 2.4.4. Microstructure

The microstructure of the chickpea–quinoa snacks at 50% (*w*/*w*), without (50CPF50QF) and with (50CPF50QF+I) inulin, was observed by means of a field emission scanning electron microscope (FESEM) (JEOL, model JSM-5410, Japan) to assess the influence of the inulin incorporation. Both samples were fixed on copper stubs, and cryofractured through immersion in liquid nitrogen, in order to observe the cross-sections. Samples were coated with platinum, and observed using an accelerating voltage of 2 kV.

#### 2.4.5. Sensory Evaluation

Sensory analysis was realized by a panel of 44 untrained panelists (18–65 years of age; 42% male, 58% female) who regularly consumed cracker-type snacks. Each panelist evaluated three samples (100 CPF and 50CPF50QF (without inulin), and 50CPF50QF+I (with inulin)) and scored the odor, color, texture, crispiness/crunchiness, taste, and overall acceptability on a 7-point hedonic scale (1 = dislike extremely, to 7 = like extremely; UNE-ISO 4121:2006, [30]). Additional questionnaire space was also provided, in which panelists could provide unstructured commentary about each sample. A final question regarding the buying intention of these products was formulated. The sensory evaluation was approved by the Ethics Committee for Research Activities at the Polytechnic University of Valencia (reference P02_22_06_2022). The samples were presented monadically, and the presentation order was randomized across the panelists. Mineral water was provided as a palate cleanser.

### 2.5. Statistical Analysis

The results obtained using the different methods were statistically analyzed using Statgraphics Centurion XVI version 16.1 software (StatPoint Technologies, Inc., Warrenton, VA, USA), using the variance analysis (ANOVA) with a significance level of 95%.

## 3. Results

### 3.1. Flour Characterization

#### 3.1.1. Proximate Composition and Mineral Content

The proximal chemical composition of the quinoa and chickpea flours (QF and CPF) are summarized in Table 2. This also includes the most representative macrominerals and microminerals. The inulin composition, as described by the provider, is included, as well. For the QF, the water content was found to be within the previously reported values of 11.64% [31] or 10.09–12.23% [32]. The protein content for the QF was higher than that reported in other references (17.42–19.59%, [31,32]). The fat content obtained is within a previously reported range of 4.87–6.06% [33] or 5.47% [28]. The ash content varies according to the consulted sources, due not only to the influence of the variety, but also to the method used to obtain the flour, as the minerals are found mainly in the outer layers of the pseudocereal grain. Thus, for the different varieties of quinoa studied by [32], the values obtained are in the range of 2.12 to 5.21%, while for [34], they are between 3.66 and 4.41%. On the other hand, according to [33], the values range between 1.74 and 2.63, and according to [35], they vary between 0.41 and 3.16%, depending on the different particle size fractions. Therefore, the obtained values for the ash content (Table 2) can be considered within some previously reported values. Finally, regarding the carbohydrate composition, the value obtained by difference is somewhat lower, compared to those obtained by [32] and by [31], which ranged between 66.63 and 72.84%. In general, the chemical composition of quinoa flour depends on the genetic variability and environmental conditions [32], just as it is also influenced by the particle size [35]. For the CPF, the obtained results are closed referred by [36], except for the crude fiber, which is much lower (3.8%).

The most abundant macromineral was phosphorus for both the QF (325 mg/100 g) and CPF (337 mg/100 g). Other abundant macrominerals were potassium, calcium, and magnesium, which showed similar values for both the vegetable flours, except for calcium, which was significantly higher in the CPF. The most prominent micromineral was Fe, with a higher content (5.1 mg/100 g) in the CPF; however, the QF also presented a relevant amount (3.64 mg/100 g).

#### 3.1.2. Particle Size Distribution

**Figure 1:**
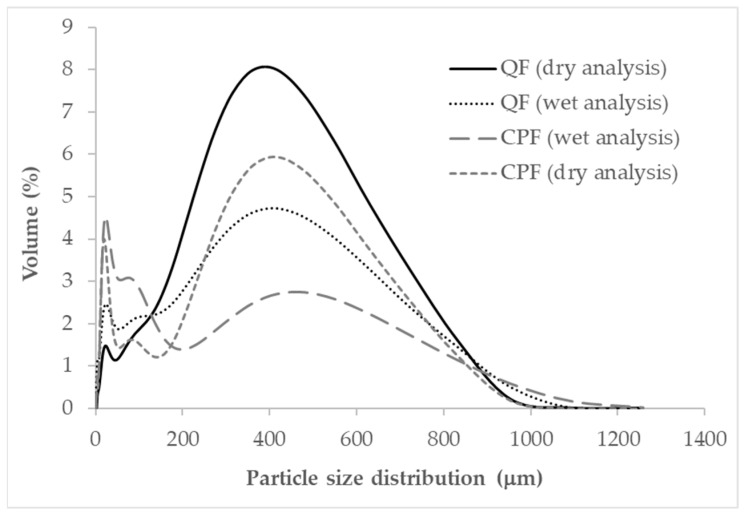
Particle size distribution of the quinoa flour (QF) and chickpea flour (CPF).

The particle size distribution provides information about the flow and bulk handling behavior of the flour, as well as its capacity (velocity), and the uniformity of its water absorption [37]. The particle size will therefore affect the behavior of the formulations that are developed from this flour, the uniformity of the mass of the final product in which they are used [38] and, consequently, sensory characteristics such as the appearance, taste, and texture. It will also influence the susceptibility to cohesion during transport and handling. Figure 1 shows the particle size distribution (dry and wet analysis) obtained for the whole quinoa flour and the chickpea flour used in the snack production studied (mean values). A distribution can be seen, with a smaller population on the left and, on the right, another larger one, which indicates a certain heterogeneity. This is logical, as it was not our intention to remove the entire outer covering of the grain.

The mean particle size, expressed as the mean diameter of the equivalent volume (D [4.3]), was 273 μm for the QF, and 192 for the CPF (Table 3). The obtained values differ from those reported by [39] for the quinoa flour obtained from the seed, with a lower mean diameter, but a more heterogenous particle size distribution (span value). Both the variety in, and the method of milling and conditioning of the obtained flour (information not provided in this previous work) could be responsible for these differences. The flour characterized in this work was obtained via sieving through a mesh size of 250 μm, so the larger particle size and, therefore, lesser surface area available for the formation of cohesive forces, could prevent the formation of lumps during handling (better fluidity). However, it can also be responsible for poorer hydration. It can be observed in Table 3 that the average particle size (expressed as the mean diameter of the equivalent volume) was significantly lower for the CPF. Different starch morphology could be responsible for the significantly higher particle size of the QF (wet analysis). Furthermore, the uniformity in the particle size distribution (span) was higher in the CPF (from industrial milling) than the QF (obtained after milling and sieving). The greater presence of fiber in the CPF (13.6 (0.7)%), compared to 2.2 (0.2)% in the QF, could be responsible for the greater heterogeneity (span 9.6 for the CPF, and 5.0 for the QF).

#### 3.1.3. Functional Properties

Using plant proteins in the food industry becomes a challenge, as several changes may occur in the technofunctional properties of the meat or animal proteins involved in the characteristics of products, such as the appearance, texture, and mouthfeel. Thus, understanding protein behavior, and changes through the process chain, is imperative to optimizing and reducing production hurdles when working with plant proteins, such as chickpeas or quinoa. Table 4 shows the average values for the assessed functional properties, which are greatly affected by the composition of the flour, and are related to the interactions between its components.

The water retention capacity (WHC) allows the evaluation of the flour’s ability to retain water under a gravitational centrifugal force; macromolecules such as carbohydrates and proteins, by offering hydrophilic sidechains, increase this retention [33]. Authors such as [40,41] report a mean value similar to that obtained in the present study, and others such as [34] also reached comparable values, and studied how the WHC increases with a reduction in the particle size (due to an increase in the surface-to-volume ratio), and as the temperature increases from 25 to 70 °C (as a consequence of starch gelatinization and protein denaturation). Thus, the assessed QF and CPF, with a starch content close to 60%, and a high protein content (22%), have a high WHC, higher than that found for cereals such as wheat (0.84 ± 0.06) [32]. It should be noted that the amylopectin found in quinoa starch has short chains, and provides this pseudocereal with a higher viscosity and water-holding capacity, and higher swelling properties, compared to other cereals, such as wheat, barley, or maize [32]. The fat absorption capacity (FAC) gives information about the interaction between the lipids and the nonpolar sidechain of the amino acids present in the flour and, therefore, the capacity to bind the oil, which will determine the mouthfeel and flavor retention of the product [33,34]. The result achieved in this study agrees with that obtained by other authors [34], but is somewhat lower than that reported by [16], although the oil used in that case was soybean. In any case, it is promising, being in the same order as those registered for cereals such as rice [16], which are widely used in products intended for the population with gluten intolerance, where fat absorption is desirable, such as bakery products or meat derivatives. In summary, the assessed quinoa and chickpea flours could be good candidates for the formulation of soups, sauces, doughs, fermented or otherwise, and baked goods, by providing viscosity, and helping to achieve a smooth texture [42]. The foaming ability (FC) is the property of proteins to form a thin and resistant film at a liquid–gas interface, by means of force and mechanical agitation movements. The protein must migrate to the interface, adsorb, and reorganize, in order to form a rigid and stable viscoelastic film that surrounds the gas bubble. A homogeneous distribution of small bubbles provides softness and lightness, as well as an increase in the dispersion and perceptibility of the aromas. This property will be influenced not only by the amount of protein present, but also by its solubility, depending on the pH and the degree of protein denaturation that may take place during processing. The results obtained are higher than those obtained by [42] for quinoa flour (9.0% for the FC and 2.0% for the FS), and those reported by [43] for wheat flour (taken as a reference for bakery and confectionery products), and rice flour (without gluten and with a high starch content, such as quinoa) with 12.9% and 3.5% for the FC, and 1.9% and 0.98% for the FS, respectively. On the other hand, the stability of the foam also turned out to be high. In summary, the evaluated QF and CPF could be used in baking and confectionery, where a certain sponginess is sought in the final product [44], and in the preparation of other products, such as ice cream or milkshakes [42]. Concerning the swelling capacity (SC), which refers to the ability to increase the volume of the product in the presence of water, this is directly related to the water absorption capacity, and is also characterized by being a functional property of proteins, fundamental in the preparation of viscous foods, such as doughs and baked goods, where a good protein–water interaction is required [44]. Contrary to expectations, the studied QF presented a low swelling capacity, compared to other authors [33], and the CPF showed a significant lower one (*p* < 0.05) than the QF.

### 3.2. Characterization of Quinoa–Chickpea Cracker-Type Snacks

#### 3.2.1. Physicochemical Analysis and Nutritional Aspects

The obtained values for the water content, water activity, final weight, and final thickness of the cracker-type snacks (Table 5) did not show significant differences (*p* < 0.05), independent of the chickpea flour’s replacement with quinoa or inulin. The water activity was below 0.6, which generally inhibits bacterial, yeast, and mould growth, as well as chemical and enzymatic changes in food [45].

**Figure 2:**
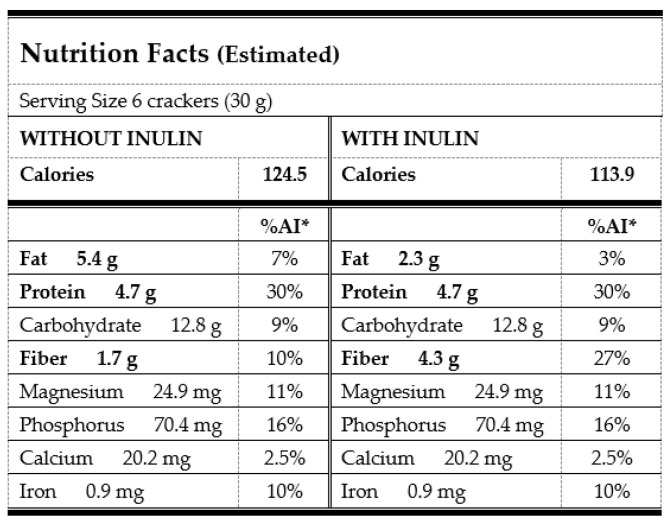
Nutritional fact label for the 50CPF50QF snacks. * Adequate intake (AI) for children aged 6–9 years (protein), 4–17 years (fat), 7–10 years (fiber and carbohydrates), and 6–10 years (minerals). AI is the average nutrient level consumed daily by a typical healthy population that is assumed to be adequate for the population’s needs.

Based on a single serving size of 30 g (Figure 2), the 50CPF50QF from the trial may contain 124.5 (with more fat), and 113.9 calories (with inulin), compared to the 120–160 calories in the existing cracker products on the market. The final energy density may decrease by 8.5% when inulin is used as a fat replacer at a 75% (*w*/*w*) level, but the energy coming from fat is considerably diminished, as the fat is reduced by 57%. As expected, there is also a remarkable increase in the fiber intake as a result of the inulin incorporation, reaching 27% of the average requirement of 16 g/day for children aged 7–10 years [5]. The final product may reach the claim of “source of fiber”, according to Regulation (EC) No 1924/2006 [46], as values are over 3% *w*/*w*. Some evidence suggests that fiber intake may play a role in the prevention and treatment of childhood obesity and constipation [47]. The protein level in these cracker-type snacks is similar to, or slightly higher than, that in commercial products, but a good balance of essential amino acids is expected, based on the quinoa and chickpea data from the literature, as commented on in the introduction section.

Concerning the mineral contribution, the magnesium, phosphorus, calcium, and iron intakes were found to be, respectively, approximately 6, 9, 1.5, and 6% of the adequate values (AI) from the European Food Safety Authority for children [5]. Those of magnesium and phosphorus are especially notable, as they are above the amount that can be found in similar products on the market. This could help to prevent insulin resistance in obese children [14].

#### 3.2.2. Snack Firmness

The obtained values for the snack firmness are shown in Table 6. The breaking point ranged between 24 and 44 N when inulin was not used, and between 40 and 85 N when vegetable oil was substituted at 75% (*w*/*w*) by this fiber. Different behavior could be observed with the amount of quinoa flour, depending on the sunflower oil substitution with inulin. Contrary to the expected results, the firmness was only significantly increased (*p* < 0.05) when the chickpea flour was replaced by quinoa flour at the 50% (*w*/*w*) level (for the non-inulin crackers), and significantly decreased with the quinoa amount (for the inulin crackers). Previous studies on cookies [48] and biscuits [49] found that the use of quinoa flour led to more compact products, with harder characteristics. These authors used wheat flour, while chickpea flour was the basis of the cracker-type snacks assessed in the present work. It is known that, together with the baking conditions, the protein content of the raw materials plays an important role in the final product texture, influencing the hardness [50], but no specific trend related to the protein content of the used flours and breaking point could be recorded. However, the obtained results reveal that, as expected, the use of inulin in the product formula led, in all cases, to a significantly higher resistance to fracture, as a result of its water-binding properties.

**Figure 3:**
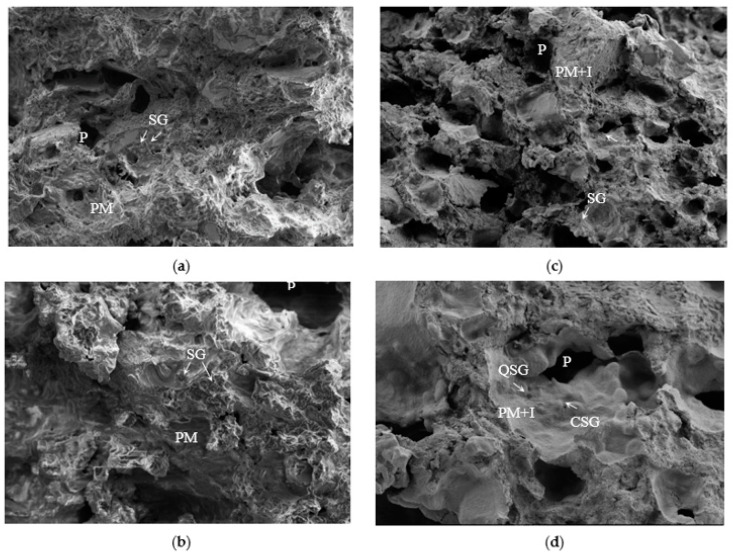
SEM observations of the 50CPF50QF cracker-type snacks (magnification 100× on the top and 300× on the bottom): (**a**,**b**) without inulin; and (**c**,**d**) with inulin. SG, starch granule; QSG, quinoa starch granule; CSG, chickpea starch granule; P, pore; PM, protein matrix; PM+I, protein–inulin matrix.

Microstructural observations (Figure 3) allowed us to see that the crackers present an alveolate morphology, with the presence of inulin favoring the appearance of a greater number of pores (P) distributed homogeneously throughout the structural profile. This could support the increased hardness observed in this formulation (50CPF50QF+I, Figure 3). Likewise, the presence of this fiber seems to assist in the formation of a more compact protein-fibrous matrix, with the chickpea starch granules (more heterogeneous in size, as described by [51]) and quinoa starch granules (smaller and more homogeneous, as reported by [32]) embedded in it. Again, this could support the increased fracture strength observed in the inulin formulations.

#### 3.2.3. Optical Properties

**Figure 4:**
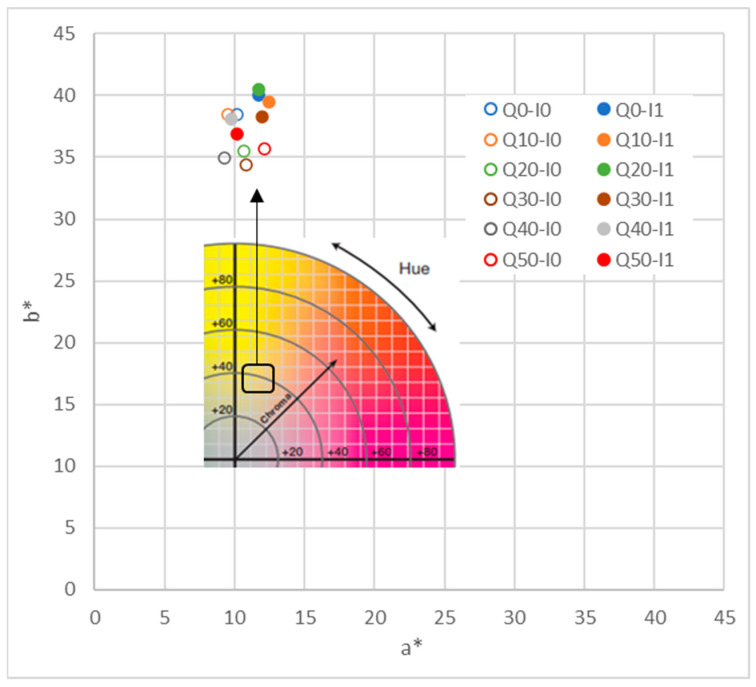
Chromatic plane a*–b* for all the snack formulations.

Color is one of the attributes that determines the acceptability of baked products [52]. It is influenced by the raw materials’ color but, mainly, the color development in such products is the result of Maillard or caramelization reactions that take place between the reducing sugars and amino acids present in the flours and other components [50], which are dependent on the water activity of the product, and the internal temperature [53]. The lightness (L*_ab_) and the psychometric coordinates (C*_ab_ and h*_ab_) of the assessed snacks are summarized in Table 6. The calculated values for the browning index (BI) are included, as well. For a better visualization, the chromatic plane a*–b* is shown in Figure 4. It can be observed that, in general, the color is highly affected neither by the inulin nor by the quinoa incorporation in the formulation, as all the developed products showed a yellow–beige color. Nevertheless, an analysis of variance was performed, considering, individually, the factors of inulin presence or quinoa percentage (a multifactor analysis did not give relevant additional information). The significant letters are included in Table 6.

As a global tendency, it seems that quinoa flour, when used above 20% (*w*/*w* flour), reduces the snack lightness, chrome, and hue angle, causing it to become darker and browner. Similar results were obtained by [49,54] when the amount of quinoa flour used in the evaluated products increased (in gluten-free cookies and biscuits, respectively). This could be not only a consequence of the characteristic color of the used raw materials (more yellow for chickpea, and beiger for quinoa), but also of the higher protein content of quinoa flour, which may increase the Maillard browning during baking [49,55]. Concerning the sunflower oil substitution with inulin powder, the obtained results reveal that the browning index (BI) and the color saturation (C*_ab_ values) significantly increased (*p* < 0.05) independent of the flour mixture used in the formulation. Inulins are thermally stable in short food-processing steps, but some hydrolysis may occur; in fact, inulin undergoes the Maillard reaction and caramelization, meaning that bakery products made with inulin have a nice, brown color [56].

#### 3.2.4. Sensory Evaluation

**Figure 5:**
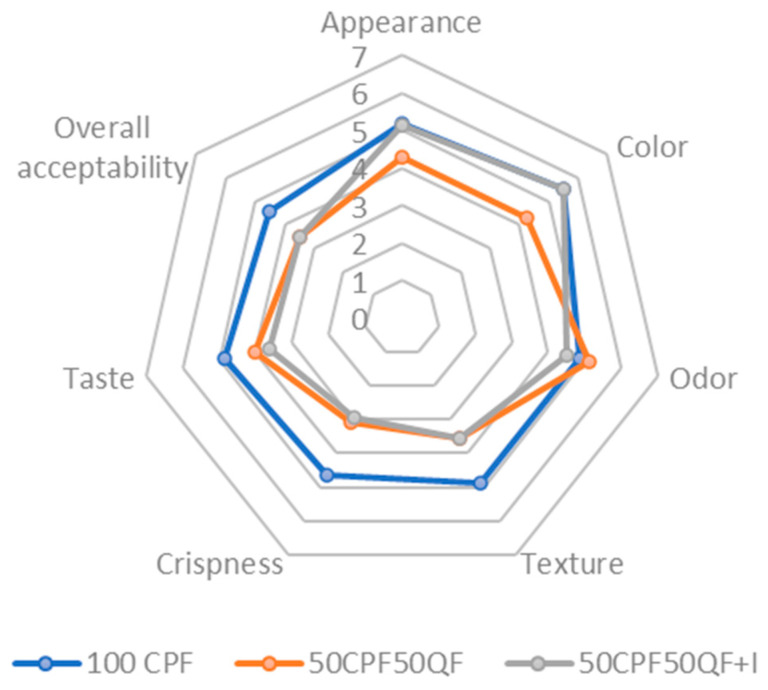
The sensory parameters of the 100 CPF, 50CPF50QF, and 50CPF50QF+I samples.

The scores for the sensory properties of the cracker-type snacks are summarized in Figure 5. Based on the commented results, especially those concerning the mechanical properties, only three formulations were tested, to assess the consumer perception when substituting chickpea flour with a quinoa–chickpea flour mix at 50% (*w*/*w*), and the effect of using inulin instead of sunflower oil. The panelists were asked to evaluate the appearance, color, odor, texture, crispness, taste, and overall acceptability. The obtained data showed a clear preference for those crackers based only on chickpea flour (the control sample), with higher scores for all the assessed attributes, except the odor, which was slightly lower. The results for the empirical firmness (Section 3.2.2) and sensorial crispness for this sample are in accordance, decreasing when the quinoa flour is used. Both the crispness and taste seem to have determined the higher overall acceptability obtained by the chickpea crackers (4.5 (1.2)). A slight bitterness seemed to be responsible for the lower scores for the taste attribute, although they was acceptable; similar results were observed by [57] when using quinoa seeds to produce bread. This tendency remained when substituting 75% (*w*/*w*) of sunflower oil with inulin (50CPF50QF+I) but, in this case, the color, appearance, and odor obtained better scores, close to those of the chickpea snacks (100 CPF).

The panelists also stated that they would regularly consume the cracker formulated only with chickpea flour and would add it to their shopping cart, and as for the formulations with quinoa, they were considered to be a very interesting healthy alternative both for children and adults, although the texture should be improved to achieve a crisp product. Regarding the taste, panelists did not detect differences between the sample with inulin and the one that did not have this fiber in its composition.

## 4. Conclusions

Taking into account all of the assessed parameters, it may be concluded that cracker-type snacks made using 50% quinoa flour (*w*/*w* total flour used), and with 75% of their high oleic sunflower oil substituted by chicory inulin, could be an interesting alternative for children as, in addition to offering a very interesting nutritional contribution (a balance of essential amino acids, and healthy fats, and a source of fiber and minerals), its energy density decreases by 5.4% (244.2 and 231.0 Kcal/100 g for the 50CPF50QF and 50CPF50QF+I formulations, respectively, calculated using the Atwater system conversion factors, in accordance with FAO recommendations). More relevant is the reduction in the energy intake from fat (57%). However, it is necessary to continue with further research, to achieve a crunchier texture.

## Figures and Tables

**Table 1:** Formulations tested in the preparation of crackers.

	g/100 g Flour	
Sample	CPF	QF
100CPF	100	0
90CPF10QF	90	10
80CPF20QF	80	20
70CPF30QF	70	30
60CPF40QF	60	40
50CPF50QF	50	50

**Table 2:** Proximate composition and individual mineral content of the quinoa and chickpea flours. Mean value (standard deviation).

		QF	CPF	I **
Water (g/100 g)		11.323 (0.109)	10.05 (0.12)	0
Protein (g/100 g)		22.2 (0.7)	22.32 (1.02)	5.7
Fat (g/100 g)		5 (2)	4.9 (0.5)	-
Ash (g/100 g)		2.56 (0.07)	2.61 (0.08)	-
**Individual Mineral Content (mg/100 g)**	Fe	3.64 (0.09)	5.1 (0.8)	-
	Cu	0.53 (0.08)	0.73 (0.14)	-
	Zn	3.3 (1.3)	2.9 (0.2)	-
	Ca	58.88 (1.07)	130 (14)	-
	Mg	122 (2)	112.0 (0.3)	-
	P	325 (5)	337 (4)	-
	K	252 (4)	266 (5)	-
	Na	17 (7)	3.52 (0.12)	-
	B	0.75 (0.02)	0.80 (0.03)	-
	Mn	1.49 (0.04)	1.81 (0.02)	-
	Mo	0.547 (0.102)	0.37 (0.12)	-
	Se	ND	ND	-
Crude fiber (g/100 g)		2.2 (0.2)	13.6 (0.7)	77.5
DC * (g/100 g)		58.901	61.5	-
Energy (kcal/100 g)		369.40	379.38	179

* Digestible carbohydrates by difference; ND, none detected. ** As described by the supplier.

**Table 3:** Mean value (standard deviation) for the particle size parameters of the quinoa and chickpea flours (^DA^: dry analysis, ^WA^: wet analysis).

	D [4.3] (µm)	d (0.1) (µm)	d (0.5) (µm)	d (0.9) (µm)	Span
QF^DA^	273 (4) ^aA^	22.1 (0.5) ^aA^	259 (4) ^aA^	547 (4) ^aA^	2.03 (0.02) ^bB^
CPF^DA^	192 (16) ^bA^	11.0 (0.4) ^bA^	93 (26) ^bA^	519 (19) ^bA^	5.6 (1.3) ^aB^
QF^WA^	189 (6) ^aB^	5.3 (0.2) ^bB^	99 (6) ^aB^	504 (13) ^aB^	5.0 (0.2) ^bA^
CPF^WA^	138 (8) ^bB^	10.1 (0.2) ^aB^	45.4 (1.7) ^bB^	446 (28) ^bB^	9.6 (0.4) ^aA^

D [4.3], d(0.1), d(0.5), d(0.9), and span represent, respectively, the equivalent volume mean particle diameter, 10%, 50%, and 90% of all particles finer than this size, and the width of the size distribution. ^a,b^ For each type of analysis (DA o WA), the means with different superscripts within the columns are significantly different (*p* < 0.05). ^A,B^ For each type of flour (QF or CPF), the means with different superscripts within the columns are significantly different (*p* < 0.05).

**Table 4:** Mean value (standard deviation) of the functional properties of the quinoa and chickpea flours (WHC, water holding capacity; FAC, oil absorption capacity; FC, foaming capacity; FS, foaming stability; SC, swelling capacity).

	WHC (g/g)	FAC (g/g)	FC (%)	FS (%)	SC (mL/g)
**QF**	1.70 (0.15) ^a^	1.4 (0.3) ^a^	16.6 (1.2) ^b^	77 (8) ^a^	2.82 (0.15) ^a^
**CPF**	1.98 (0.2) ^b^	1.25 (0.04) ^b^	28.3 (1.2) ^a^	19.8 (0.5) ^b^	1.12 (0.02) ^b^

^a,b^ The means with different superscripts within the columns are significantly different (*p* < 0.05).

**Table 5:** Physicochemical properties of cracker-type snacks with and without inulin.

	Without Inulin (I = 0)				With Inulin (I = 1)			
Sample	Water Content (g/g)	Weight (g)	Thickness (mm)	a_w_	Water Content (g/g)	Weight (g)	Thickness (mm)	a_w_
100CPF	0.120 (0.015) ^aA^	5.0 (0.3) ^aA^	2.000 (<0.001) ^aA^	0.490 (0.016) ^aA^	0.1200 (0.0013) ^aA^	5.00 (0.15) ^aA^	2.0 (0.3) ^aA^	0.50 (0.02) ^aA^
90CPF10QF	0.120 (0.012) ^aA^	5.0 (0.3) ^aA^	2.0 (0.4) ^aA^	0.500 (0.012) ^aA^	0.130 (<0.001) ^aA^	5.000 (<0.001) ^aA^	3.000 (<0.001) ^aA^	0.540 (<0.001) ^aA^
80CPF20QF	0.140 (<0.001) ^aA^	6.000 (<0.001) ^aA^	2.000 (<0.001) ^aA^	0.550 (<0.001) ^aA^	0.13 (0.05) ^aA^	5.0 (0.5) ^aA^	3.0 (0.2) ^aA^	0.550 (0.012) ^aA^
70CPF30QF	0.140 (0.003) ^aA^	5.00 (0.07) ^aA^	3.0 (0.8) ^aA^	0.5400 (0.0008) ^aA^	0.140 (0.005) ^aA^	5.0 (0.6) ^aA^	3.0 (0.6) ^aA^	0.540 (0.004) ^aA^
60CPF40QF	0.140 (0.007) ^aA^	5.000 (0.112) ^aA^	2.000 (<0.001) ^aA^	0.550 (0.005) ^aA^	0.130 (0.011) ^aA^	5.0 (0.4) ^aA^	2.0 (0.3) ^aA^	0.530 (0.012) ^aA^
50CPF50QF	0.14 (0.04) ^aA^	5.0 (0.7) ^aA^	3.0 (0.7) ^aA^	0.530 (0.006) ^aA^	0.10 (0.04) ^aA^	6.00 (0.6) ^aA^	3.0 (0.8) ^aA^	0.490 (0.007) ^aA^

The values are reported as the means (standard deviation) of three replicates. ^a^ The means with different superscripts within the columns are significantly different (*p* < 0.05). ^A^ The means with different superscripts within the rows for the same parameter are significantly different (*p* < 0.05).

**Table 6:** The optical and mechanical properties of the cracker-type snacks with and without inulin (L*_ab_, luminosity; C*_ab_, chrome; h*_ab_, hue angle; BI, browning index; F, firmness).

	Without Inulin (I = 0)					With Inulin (I = 1)				
Sample	L*_ab_	C*_ab_	h*_ab_	BI	F (N)	L*_ab_	C*_ab_	h*_ab_	BI	F (N)
100CPF	65.0 (0.7) ^aA^	40.0 (0.4) ^aB^	76.0 (1.7) ^aA^	103 (4) ^abB^	29.03 (1.12) ^bcB^	64 (2) ^aA^	42.0 (0.9) ^aA^	74 (3) ^bA^	112 (9) ^bA^	85 (5) ^aA^
90CPF10QF	63 (3) ^aA^	40 (3) ^aB^	75 (2) ^aB^	104 (3) ^abB^	24 (2) ^cB^	59 (3) ^cdB^	41.0 (0.8) ^aA^	72 (3) ^bA^	124 (8) ^aA^	60 (17) ^bA^
80CPF20QF	58.16 (1.06) ^cA^	37 (2) ^bB^	73.4 (1.9) ^bA^	109 (10) ^abB^	32 (8) ^bB^	59.7 (1.2) ^cdA^	42.2 (0.9) ^aA^	73.84 (1.14) ^abA^	126 (7) ^aA^	56 (15) ^bA^
70CPF30QF	55.9 (0.4) ^dB^	36 (3) ^bB^	72.6 (1.2) ^bcA^	111 (11) ^aB^	31 (6) ^bB^	57.6 (0.) ^dA^	40.0 (0.8) ^bA^	72.7 (1.3) ^bA^	123 (5) ^aA^	46 (12) ^bA^
60CPF40QF	59.1 (0.8) ^bB^	36.2 (1.2) ^bB^	75.2 (0.5) ^aA^	103 (4) ^bB^	28 (4) ^bcB^	61.8 (0.9) ^bA^	39.3 (1.4) ^bA^	75.678 (1.012) ^aA^	108 (7) ^bA^	48 (4) ^bA^
50CPF50QF	58.4 (0.8) ^bcB^	37.7 (0.8) ^bA^	71.2 (1.8) ^cB^	110 (6) ^aA^	44 (9) ^aB^	60.3 (0.8) ^bcA^	38.3 (0.5) ^cA^	74.6 (1.6) ^abA^	108 (3) ^bA^	40 (6) ^bA^

The values are reported as the means (standard deviation) of three replicates. ^a–d^ The means with different superscripts within the columns are significantly different (*p* < 0.05). ^A,B^ The means with different superscripts within the rows for the same parameter are significantly different (*p* < 0.05).

## Data Availability

Data sharing is not applicable to this article.

## References

[1] WHO (2021). World Health Organisation. Obesity and Overweight. https://www.who.int/news-room/fact-sheets/detail/obesity-and-overweight.

[2] Aranceta-Bartrina J., Gianzo-Citorese M., Pérez-Rodrigo C. (2020). Prevalence of overweight, obesity and abdominal obesity in the Spanish population aged 3 to 24 years. The ENPE study. Rev. Esp. Cardiol..

[3] Block G. (2004). Foods contributing to energy intake in the US: Data from NHANES III and NHANES 1999–2000. J. Food Compost. Anal..

[4] AESAN (2019). Informe del Comité Científico de la Agencia Española de Seguridad Alimentaria y Nutrición (AESAN) sobre Ingestas Nutricionales de Referencia para la población española (AESAN-2019-003). Rev. Com. Cient..

[5] European Food Safety Authority (EFSA) Dietary Reference Values for the European Union. https://www.efsa.europa.eu/en/topics/topic/dietary-reference-values.

[6] Shoaib M., Shehzad A., Omar M., Rakha A., Raza H., Sharif H.R., Shakeel A., Ansari A., Niazi S. (2016). Inulin: Properties, health benefits and food applications. Carbohydr. Polym..

[7] Bayarri S., González-Tomás L.U., Hernando I., Lluch M.A., Costell E. (2011). Texture perceived on inulin-enriched low-fat semisolid dairy desserts. Rheological and structural basis. J. Texture Stud..

[8] Nanduri K.R., Hirich A., Salehi M., Saadat S., Jacobsen S.E., Khan M.A., Boër B., Özturk M., Clüsener-Godt M., Gul B., Breckle S.-W. (2019). Quinoa: A new crop for harsh environments. Sabkha Ecosystems.

[9] Asati R., Tripathi M.K., Tiwari S., Yadav R.K., Tripathi N. (2022). Molecular Breeding and Drought Tolerance in Chickpea. Life.

[10] López M.J., Mohiuddin S.S. (2023). Biochemistry, Essential Amino Acids.

[11] Elsohaimy S.A., Refaay T.M., Zaytoun M.A.M. (2015). Physicochemical and functional properties of quinoa protein isolate. Ann. Agric. Sci..

[12] FAO/WHO (1991). Protein Quality Evaluation: Report of a Joint FAO-WHO Expert Consulation.

[13] Ren G., Teng C., Fan X., Guo S., Zhao G., Zhang L., Liang Z., Qin P. (2023). Nutrient composition, functional activity and industrial applications of quinoa (*Chenopodium quinoa* Willd.). Food Chem..

[14] Huerta M.G., Roemmich J.N., Kington M.L., Bovbjerg V.E., Weltman A.L., Holmes V.F., Patrie J.T., Rogol A.D., Nadler J.L. (2005). Magnesium deficiency is associated with inulin resistance in obese children. Diabetes Care.

[15] Cuadrado-Soto E., López-Sobaler A.M., Jiménez-Ortega A.I., Aparicio A., Bermejo L.M., Hernández-Ruiz A., Lara-Villoslada F., Leis R., Martínez de Victoria M., Moreno J.M. (2020). Usual Dietary Intake, Nutritional Adequacy and Food Sources of Calcium, Phosphorus, Magnesium and Vitamin D of Spanish Children Aged One to <10 Years. Findings from the EsNuPI Study. Nutrients.

[16] Di Cairano M., Condelli N., Caruso M.C., Marti A., Cela N., Galgano F. (2020). Functional properties and predicted glycemic index of gluten free cereal, pseudocereal and legume flours. LWT-Food Sci. Technol..

[17] Acevedo Martinez K.A., Yang M.M., de Mejia E.G. (2021). 2021 Technological properties of chickpea (*Cicer arietinum*): Production of snacks and health benefits related to type-2 diabetes. Compr. Rev. Food Sci. Food Saf..

[18] AOAC (2000). Official Methods of Analysis of AOAC International.

[19] Fukalova Fukalova T., García-Martínez M.D., Raigón M.D. (2022). Nutritional Composition, Bioactive Compounds, and Volatiles Profile Characterization of Two Edible Undervalued Plants: *Portulaca oleracea* L. and *Porophyllum ruderale* (Jacq.) Cass. Plants.

[20] (2009). Particle Size Analysis-Laser Diffraction Methods.

[21] Heywood A.A., Myers D.J., Bailey T.B., Johnson L.A. (2002). Functional properties of low-fat soy flour produced by an extrusion expelling system. J. Am. Oil Chem. Soc..

[22] Lin C.S., Zayas J.F. (1987). Functionality of defatted corn germ proteins in a model system: Fat binding capacity and water retention. J. Food Sci..

[23] Ahn H.J., Kim J.H., Ng P.K.W. (2005). Functional and thermal properties of wheat, barley, and soy flours and their blends treated with a microbial transglutaminase. J. Food Sci..

[24] Narayana K., Narasinga Rao M.S. (1982). Functional properties of raw and heat processed winged bean (*Psophocarpus tetragonolobus*) flour. J. Food Sci..

[25] Alu’datt M.H., Rababah T., Ereifej K., Alli I., Alrababah M.A., Almajwal A., Masadeh N., Alhamad M.N. (2012). Effects of barley flour and barley protein isolate on chemical, functional, nutritional and biological properties of Pita bread. Food Hydrocolloid..

[26] Colla K., Gamlath S. (2015). Inulin and maltodextrin can replace fat in baked savoury legume snacks. Int. Food Sci. Technol..

[27] Di Cairano M., Galgano F., Tolve R., Caruso M.C., Condelli N. (2018). Focus on gluten free biscuits: Ingredients and issues. Trends Food Sci. Technol..

[28] AACC International (2010). Approved Methods of the American of Cereal Chemists.

[29] Sarıçoban C., Yılmaz M.T. (2010). Modelling the effects of processing factors on the changes in colour parameters of cooked meatballs using response surface methodology. World Appl. Sci. J..

[30] (2006). Sensory Analysis. Guidelines for the Use of Quantitative Response Scale.

[31] Contreras-Jiménez B., Torres-Vargas O.L., Rodríguez-García M.E. (2019). Physicochemical characterization of quinoa (*Chenopodium quinoa*) flour and isolated starch. Food Chem..

[32] Valdez-Arana J.C., Steffolani M.E., Repo-Carrasco-Valencia R., Pérez G.T., Condezo-Hoyos L. (2020). Physicochemical and functional properties of isolated starch and their correlation with flour from the Andean Peruvian quinoa varieties. Int. J. Biol. Macromol..

[33] Pellegrini M., Lucas-Gonzales R., Ricci A., Fontecha J., Fernández-López J., Pérez-Álvarez J.A., Viuda-Martos M. (2018). Chemical, fatty acid, polyphenolic profile, techno-functional and antioxidant properties of flours obtained from quinoa (*Chenopodium quinoa* Willd) seeds. Ind. Crop. Prod..

[34] Ghumman A., Mudgal S., Singh N., Ranjan B., Kaur A., Rana J.C. (2021). Physicochemical, functional and structural characteristics of grains, flour and protein isolates of Indian quinoa lines. Food Res. Int..

[35] Ahmed J., Thomas L., Arfat Y.A. (2019). Functional, rheological, microstructural and antioxidant properties of quinoa flour in dispersions as influenced by particle size. Food Res. Int..

[36] Hussein H., Awada S., El-Sayed I., Ibrahima A. (2020). Impact of chickpea as prebiotic, antioxidant and thickener agent of stirred bio-yoghurt. Ann. Agric. Sci..

[37] Albors A., Raigon M.D., Garcia-Martinez M.D., Martin-Esparza M.E. (2016). Assessment of techno-functional and sensory attributes of tiger nut fresh egg tagliatelle. LWT-Food Sci. Technol..

[38] Martín-Esparza M.E., Bressi G.B., Raga A., Albors A., Cárcel J.A., Clemente G., García-Pérez J.V., Rosselló C. (2018). Technological and nutritional aspects of gluten-free pasta based on chickpea flour and tiger nut flour. Proceedings of the 21st International Drying Symposium.

[39] Alonso-Miravalles L., Zannini E., Bez J., Arent E.K., O’Mahony J.A. (2020). Physical and flow properties of pseudocereal-based protein-rich ingredient powders. J. Food Eng..

[40] Shi D., Fidelis M., Ren Y., Stone A.K., Ai Y., Nickerson M.T. (2019). The functional attributes of Peruvian (Kankolla and Blanca juli blend) and Northern quinoa (NQ94PT) flours and protein isolates, and their protein quality. Food Res. Int..

[41] Martínez-Preciado A.H., Ponce-Simental J.A., Schorno A.L., Contreras-Pacheco M.L., Michel C.R., Rivera-Ortiz K.G., Soltero J.F.A. (2020). Characterization of nutritional and functional properties of “Blanco Sinaloa” chickpea (*Cicer arietinum* L.) variety, and study of the rheological behavior of hummus pastes. J. Food Sci. Technol..

[42] Ogungbenle H.N. (2003). Nutritional evaluation and functional properties of quinoa (*Chenopodium quinoa*) flour. Int. J. Food Sci. Nutr..

[43] Chandra S., Samsher S. (2013). Assessment of functional properties of different flours. Afr. J. Agric. Res..

[44] García Y., Cabrera D., Ballestas J.A., Campo M.J. (2019). Effect of different thermal treatments on the tecfunctional properties of white bean flour (*Phaseolus lunatus* L.) and the determination of its potential agri-food use. INGE CUC.

[45] Fontana A., Barbosa-Cánovas G.V., Fontana A.J., Schmidt S.J., Labuza T.P. (2020). D: Minimum Water Activity Limits for Growth of Microorganisms. Water Activity in Foods, Fundamentals and Applications.

[46] European Commission (2006). Regulation (EC) No 1924/2006 of the European Parliament and of the Council of 20 December 2006 on nutrition and health claims made on foods. Off. J. Eur. Union.

[47] Anderson J.W., Baird P., Davis R.H., Ferreri S., Knudtson M., Koraym A., Waters V., Williams C.L. (2009). Health benefits of dietary fiber. Nutr. Rev..

[48] Demir M.K., Kilinç M. (2017). Utilization of quinoa flour in cookie production. Int. Food Res. J..

[49] Moawad E.M.M., Rizk I.R.S., Kishk Y.F.M., Youssif M.R.G. (2019). Effect of substitution of wheat flour with quinoa flour on quality of pan bread and biscuit. AUJASCI Arab Univ. J. Agric. Sci..

[50] Di Cairano M., Condelli N., Caruso M.C., Cela N., Tolve R., Galgano F. (2021). Use of underexploited flours for the reduction of glycaemic index of gluten-free biscuits: Physicochemical and sensory characterization. Food Bioprocess Technol..

[51] Ma Z., Boye J.I., Simpson B.K., Prasher S.O., Monpetit D., Malcolmson L. (2011). Thermal processing effects on the functional properties and microstructure of lentil, chickpea, and pea flours. Food Res. Int..

[52] Zucco F., Borsuk Y., Amtfield S.D. (2011). Physical and nutritional evaluation of wheat cookies supplemented with pulse flours of different particle sizes. LWT-Food Sci. Technol..

[53] Purlis E. (2010). Browning development in bakery products—A review. J. Food Eng..

[54] Brito I.L., Souza E.L., Felex S.S.S., Madruga M.S., Yamashita F., Magnani M. (2015). Nutritional and sensory characteristics of gluten-free quinoa (*Chenopodium quinoa* Willd)-based cookies development using an experimental mixture design. J. Food Sci. Technol..

[55] El-Sohaimy S.A., Shehata M.G., Mehany T., Zeitoun M.A. (2019). Nutritional, Physicochemical, and Sensorial Evaluation of Flat Bread Supplemented with Quinoa Flour. Int. J. Food Sci..

[56] Tiefenbacher K.F. (2017). The Technology of Wafers and Waffles I Operational Aspects.

[57] Stikic R., Glamoclija D., Demin M., Vucelic-Radovic B., Jovanovic Z., Milojkovic-Opsenica D., Jacobsen S.E., Milovanovic M. (2012). Agronomical and nutritional evaluation of quinoa seeds (*Chenopodium quinoa* Willd.) as an ingredient in bread formulations. J. Cereal Sci..

